# B Cells in Multiple Sclerosis and Virus-Induced Neuroinflammation

**DOI:** 10.3389/fneur.2020.591894

**Published:** 2020-11-03

**Authors:** Rittika Chunder, Verena Schropp, Stefanie Kuerten

**Affiliations:** Institute of Anatomy and Cell Biology, Friedrich-Alexander University Erlangen-Nuremberg, Erlangen, Germany

**Keywords:** B cells, multiple sclerosis, neuroinflammation, central nervous system, viral infection, EBV

## Abstract

Neuroinflammation can be defined as an inflammatory response within the central nervous system (CNS) mediated by a complex crosstalk between CNS-resident and infiltrating immune cells from the periphery. Triggers for neuroinflammation not only include pathogens, trauma and toxic metabolites, but also autoimmune diseases such as neuromyelitis optica spectrum disorders and multiple sclerosis (MS) where the inflammatory response is recognized as a disease-escalating factor. B cells are not considered as the first responders of neuroinflammation, yet they have recently gained focus as a key component involved in the disease pathogenesis of several neuroinflammatory disorders like MS. Traditionally, the prime focus of the role of B cells in any disease, including neuroinflammatory diseases, was their ability to produce antibodies. While that may indeed be an important contribution of B cells in mediating disease pathogenesis, several lines of recent evidence indicate that B cells are multifunctional players during an inflammatory response, including their ability to present antigens and produce an array of cytokines. Moreover, interaction between B cells and other cellular components of the immune system or nervous system can either promote or dampen neuroinflammation depending on the disease. Given that the interest in B cells in neuroinflammation is relatively new, the precise roles that they play in the pathophysiology and progression of different neuroinflammatory disorders have not yet been well-elucidated. Furthermore, the possibility that they might change their function during the course of neuroinflammation adds another level of complexity and the puzzle remains incomplete. Indeed, advancing our knowledge on the role of B cells in neuroinflammation would also allow us to tackle these disorders better. Here, we review the available literature to explore the relationship between autoimmune and infectious neuroinflammation with a focus on the involvement of B cells in MS and viral infections of the CNS.

## Introduction

Historically, the primary focus of B cells as enhancers of autoimmunity was their exclusive ability to differentiate into plasma cells and produce autoantibodies. Over the last few decades our understanding that B cells are merely responsible for the production of autoantibodies has been challenged and antibody independent effector functions of B cells are now greatly appreciated (1–8). Based on preclinical and clinical data, mounting evidences suggest that B cells effectively collaborate with T cells to initiate and fine-tune T cell-dependent responses in the development of several autoimmune diseases (9–11). B cells are also known to act as negative sensors of autoimmunity that regulate immunological functions by suppressing T cell proliferation, secreting anti-inflammatory cytokines (12, 13) and controlling monocyte activity (14–18). Consequently, B cells have now emerged to take center stage as cells with effector as well as immunoregulatory potential.

Indeed, a large volume of literature emphasizes on the heterogenous roles of B cells in autoimmunity and peripheral inflammation, yet our understanding of the extent of B cell involvement in autoimmune neuroinflammation remains incomplete.

Neuroinflammation can be defined as a coordinated and complex interaction between CNS-resident cells and the peripheral immune system and is characterized by a host of cellular and molecular changes within the CNS (19, 20). Neuroinflammation is a prominent feature in the etiology of a number of neurological disorders and diseases including multiple sclerosis (MS), and viral encephalitis (21) where the inflammatory response is generally recognized as a disease-escalating factor (22, 23). A common denominator for neuroinflammatory disorders is the impairment of the integrity of the endothelial, epithelial, and glial brain barriers that together compartmentalize the CNS from the periphery (24–26).

Cells of the innate immune system are typically the focal point for any discussion of neuroinflammation (19, 27), while B cells are not considered as the first responders of an inflammatory insult within the CNS. However, recent evidence suggests that B cells, which are largely absent in the CNS parenchyma or sparsely present in the cerebrospinal fluid (CSF) of healthy individuals (27), rapidly accumulate in the CSF (28, 29) during neuroinflammation and their numbers increase by several folds in the CNS parenchyma or the perivascular spaces (30).

Taken together, it is only recently that the importance of B cells as multifunctional players in neuroinflammatory disorders is being acknowledged with several outstanding questions requiring elucidation (31–33).

In this article, we discuss the literature available on how B cells are involved in two different instances of neuroinflammation by highlighting their beneficial and detrimental roles in ameliorating or aggravating disease pathophysiology, respectively. On the one hand, we focus on MS, which is a classical example of autoimmune neuroinflammation and on the other hand we extend our discussion by drawing parallels between MS and virus-induced neuroinflammation with respect to the involvement of B cells.

## General Introduction to B Cell Biology

As B cells are in the focus of this review, this chapter will briefly summarize the principles of B cell biology as well as provide an overview of different B cell subsets and their main functions. B cells belong to the population of lymphocytes and they are part of the adaptive immune system. They express clonally diverse antigen recognition molecules known as immunoglobulins (Igs). Membrane-bound Ig on the surface of B cells acts as a receptor, the so-called B cell receptor (BCR), that recognizes specific antigenic epitopes.

Very briefly, the development and differentiation of a B cell begins in the bone marrow from a pro-B cell to an immature naïve B cell (34, 35). At this stage of development, B cells undergo various checkpoints including clonal deletion and receptor editing, which prevents the development of auto-reactive cells (36–38). B cells that successfully complete these checkpoints leave the bone marrow as transitional B cells (39). However, the checkpoints can be imperfect and B cells capable of self-directed autoimmune responses are common and exist as a part of the healthy immune repertoire (40, 41). An immature naïve B cell migrates into a secondary lymphoid organ where it then develops into a mature naïve B cell, expressing a BCR with single antigenic specificity (42). A mature naïve B cell can generally be divided into three further subsets: B-1 B cells, marginal zone (MZ) B cells and follicular B cells, with the B-1 B cells being further subdivided into B-1a (CD4^+^ helper T cell-dependent) and B-1b (CD4^+^ helper T cell-independent) B cells (43). B-1a cells provide protection against bacterial infections while B-1b cells function independently of T helper cells and provide adaptive immune response to polysaccharides, for instance lipopolysaccharide, and other T cell-independent antigens (44). When mature naïve B cells encounter their cognate antigen in the secondary lymphoid tissue, they become activated. While the primary signal for B cell activation is the binding of antigen to its antigen-specific receptor expressed by the B cell, secondary signals are also required. A B cell response to the antigen is successful only by the synergy between the engagement of their BCR and co-receptors like Toll-like receptors (TLRs) and CD40, which control class switching and affinity maturation in these activated B cells (45, 46). Following activation, some of these B cells—in conjunction with CD4^+^ T cell help—take part in germinal center (GC) reactions within the lymphoid follicles.

Lymphoid follicles in secondary lymphoid tissue act as a site of antigen-induced B cell proliferation and they have a complex microenvironment, which consists of immune cells, adhesion molecules and antigen-antibody complexes. GCs are specialized areas within these lymphoid follicles where B cells undergo somatic hypermutation leading to affinity maturation to eventually develop into memory B cells or antibody secreting plasma cells (47, 48). The adaptive immune system can evoke an enhanced response to a previously experienced pathogen. This response depends on memory lymphocyte populations of which memory B cells are a part. The improved responsiveness of memory B cells is attributed to class switching and high affinity BCR on their surface which they develop within the GC. However, it is important to note that memory B cells are a heterogenous population and can be further differentiated into T cell-dependent/GC-dependent memory B cells or GC-independent memory B cells (49).

## Autoimmune Neuroinflammation: a Focus on Multiple Sclerosis (MS)

### An Overview of the Disease

MS is a neuroinflammatory demyelinating disorder of the CNS in genetically predisposed individuals (50). MS is considered to be a heterogenous disease with different clinical courses depending on the subtype (51). While ~85–90% of MS patients present with a relapsing-remitting form of MS (RRMS), most of these patients develop secondary progressive disability (SPMS) in the course of the disease. The rarer form of MS is primary progressive MS (PPMS) which has an insidious disease onset and is characterized by a steady increase in neurological disability (52). The pathogenic role of inflammation in all the subtypes of MS remains undisputed (53–55), and the inflammatory reaction in MS is said to be a cumulative effect of a number of factors including cells of the innate and adaptive immune system, their mediators and effector molecules like cytokines and antibodies (56–58).

### Evidence of B Cells in MS

Despite historically being dubbed as a “T-cell mediated disease,” emerging evidence suggests that B cells contribute to MS pathogenesis in more than one way (59–62). The multifaceted roles of B cells as “shapers” in MS disease progression include antibody production, pro- and anti-inflammatory cytokine secretion and antigen presentation (32, 63, 64).

One of the earliest indications that B cells contribute to disease pathogenesis comes from the identification of persistent oligoclonal bands (OCBs) in the CSF of > 90% of all patients diagnosed with clinically definite MS (65, 66). In general, the presence of OCBs suggests abnormal intrathecal production of clonally expanded IgGs which is an indication of the pathogenic role of B cells in neuroinflammatory and infectious diseases of the CNS (67). In MS, a direct link between CSF-infiltrating B cells as the source of Igs associated with these OCBs has been established (68). Two studies have demonstrated that a significantly increased accumulation of B cells in the CSF of MS patients strongly correlates with intrathecal synthesis of IgG (69, 70). Furthermore, these B cells have been characterized to be of the IgM^−^IgD^−^ class-switched memory and plasmablast phenotypes. In line with the findings above, other studies have separately identified that clonally expanded B cells in the CSF of MS patients show evidence of somatic hypermutation and affinity maturation (71, 72). Indeed, the presence of B cells is not just restricted to the CSF but overlapping B cell populations are common between the periphery and the different CNS compartments (58, 73, 74) providing proof that clonally related B cells participate in bidirectional exchange across the brain barriers in the case of MS. In another study Ig gene repertoire sequencing of CSF and peripheral blood B cells in treatment-naïve MS patients has also revealed a clonal relationship between the B cell populations in the two compartments (75).

The involvement of both B cells and autoantibodies in MS also comes from neuropathological analysis of lesions from patients. For instance, one of the most frequent patterns in MS lesions is characterized by antibody deposition and complement activation (76). Although the presence of complement supports a pathogenic role of the antibodies in correlation with areas of demyelination (76, 77), the antigenic targets for these autoantibodies remain unclear. Moving from the detection of antibodies to B cells in autopsied CNS tissue from MS patients, immunohistochemical stainings have indicated the accumulation of B cells and plasma cells in perivascular spaces of the brain which are associated with active demyelination (78). A more recent study has revealed a prominent presence of CD20^+^ B cells in the lesions of patients with acute MS (79), indicating that B cells may be important in the overall inflammatory process and also in the early stages of the MS.

Furthermore, in the secondary progressive stages of MS, lymphoid-like B cell follicles have been detected in the inflamed meninges of up to 40% of patients (80–82). These ectopic follicles containing a complex network of B- and T cells, plasma cells as well as follicular dendritic cells (83) are preferentially localized within the subarachnoid space, attached to the pial membrane and their presence is often associated with a more aggressive disease progression (60, 81). A connection between the presence of these meningeal B cell follicular aggregates and the sustenance of B cell maturation locally within the CNS leading to a compartmentalized humoral immune response has been made (61). In addition to these ectopic B cell follicles in the leptomeninges of SPMS patients, meningeal CD20^+^ B cell infiltrates have also been reported in patients with PPMS which correlate with a higher degree of cortical demyelination (84). However, as yet there is little knowledge on the (immuno)phenotype of these B cells, their molecular characteristics or the precise role they play within these follicular structures or aggregates. Of importance are the difficulties faced in studying these aggregates because of limited availability of appropriate B cell follicle containing tissue, poor quality of tissue and technical difficulties of detecting these follicles due to the easy detachment of the meninges during autopsy (85).

A number of antigen experienced B cell clones have also been detected within the CNS parenchyma in MS patients with a chronic progressive or secondary progressive disease course (86). Furthermore, a more recent study demonstrated the presence of B cell follicles in the spinal meninges of SPMS patients that were associated with demyelination and axonal loss (87). These findings suggest that B cells are probably not just localized in the extraparenchymal tissue of the brain but also populate different areas of the CNS tissue, including the spinal cord.

Despite there being little doubt regarding the presence of B cells in the different compartments of MS patients, the precise site(s) or trigger(s) of B cell activation remain fairly speculative (31, 63). One hypothesis for their activation could be that B cells encounter their cognate antigen in the peripheral deep cervical lymph nodes—which is the site of CSF-mediated drainage of brain antigens—where they differentiate into memory B cells or plasmablasts before migrating into the CNS (31). In the inflamed CNS, these plasmablasts or memory B cells may further differentiate into plasma cells. This differentiation may be even in the absence of specific antigens but rather in an antigen non-specific manner by a polyclonal stimulus (88). For example, human herpesvirus 6 (HHV-6), which is an infectious agent implicated in the pathogenesis of MS, may be involved in polyspecific B cell activation (89, 90). One of the obvious manifestations of these antibody secreting plasma cells within the CNS is in the form of OCBs as seen in the CSF of MS patients. It may also be plausible that naïve B cells enter the CNS and are activated within the CNS (for example, by taking part in GC reactions within meningeal B cell follicles) and complete the circle of eventually differentiating into plasma cells.

To summarize, the studies mentioned above indicate that the number of B cells and their location possibly depends on the disease course and duration, with a substantial amount of variation between individual cases. It supports more careful screening of autopsied CNS tissue from MS patients with a chronic disease course with the purpose of characterizing the B cells beyond their CD20 marker. The literature strongly suggests that B cells are involved in MS and are present in all the different compartments within the CNS and in the periphery. Yet, in what ways these B cells establish themselves in the inflamed brain, where and how they are activated has not yet been clearly elucidated with only a limited number of studies addressing these questions (91–93).

### Role of B Cells in MS

The importance of different antibody-independent functions of B cells in the pathogenesis of MS is highlighted by the success story of treatment with monoclonal anti-CD20 antibodies. It has been shown that depletion of circulating B cells by the chimeric anti-CD20 monoclonal antibody rituximab effectively led to rapid reduction in gadolinium (Gd)-enhancing lesions and MRI lesion load as well as relapse activity in RRMS patients (94, 95). This anti-CD20 monoclonal antibody has also shown high efficacy in the removal of CD20^+^ B cells from the peripheral and CSF compartments (96, 97). However, the reduction of B cells in the CSF was comparatively much lower than in the periphery (30, 98, 99). Ocrelizumab, a humanized anti-CD20 antibody, has demonstrated high efficacy in reducing relapse rates in RRMS patients in different clinical trials and is also associated with lower rates of clinical and MRI progression in patients with progressive MS (100, 101).

Since plasma cells do not express CD20, they are not directly depleted by anti-CD20 therapy (96). Therefore, the decrease in disease activity following treatment of MS patients with anti-CD20 antibodies is possibly linked to one or more antibody-independent functions of B cells such as antigen presentation (to T cells) or cytokine production (32).

B cells can function as effective antigen presenting cells (APCs) when they recognize the same antigen as T cells (102), which is important for the activation of effector T cells (103). As a part of this B- and T- cell cognate interactions, the combination of co-stimulatory signals plays a key role in defining the T cell response of which the interaction between CD80/CD86 and CD28 is among the best characterized (32). One such antigen presenting potential of B cells in the context of MS comes from reports indicating that during MS disease exacerbations, the number of CD80^+^ B cells abnormally increases (59, 63, 104). Exactly what set of triggers is responsible for this upregulation of CD80 in B cells of MS patients is, however, less known. One of the possibilities is that interferon (IFN)-β induces CD80 expression (104, 105), which is a cytokine produced by innate immune cells like macrophages and non-immune cells like fibroblasts and epithelial cells. It has been shown that IFN-β therapy noticeably reduces the number of circulating CD80 B cells (59, 104) in MS patients. Secondly, ligands for TLR 1/2, 4, 7/8 are also known to induce a strong activation of B cells and upregulation of CD40 and CD80 (106). In the context of MS, TLRs indeed play a major role in the initiation of disease as well as in the triggering of relapses (107, 108). Furthermore, a variety of cytokines like IL-4 and IL-2 that are relevant in the context of MS, are also known to induce CD80 expression on B cells (109).

The potential of memory B cells from RRMS patients to activate T cells has also been demonstrated by Jelcic et al. and Harp et al. (110, 111), where T helper cells promoted B cell proliferation and differentiation, thus establishing a bidirectional B- and T cell interaction that plays a key role in MS pathogenesis (110).

Furthermore, circulating B cells of untreated MS patients exhibit an abnormal balance of pro- to anti-inflammatory cytokine responses (112–114). These abnormalities in the effector-cytokine production by MS B cells in turn also affects the myeloid as well as the T cell compartments. Of note, *in vitro* studies using B cells from MS patients demonstrate the ability of granulocyte-macrophage colony-stimulating factor (GM-CSF) expressing B cells to efficiently enhance myeloid cell pro-inflammatory responses in a GM-CSF dependent manner (115). Another example comes from anti-CD20 depletion studies where changes in the number of pro-inflammatory B cells correlated with a persistent decrease of T cell lineage pro-inflammatory responses (116). These studies have demonstrated that B cells from MS patients in comparison to healthy controls cannot only produce a myriad of pro-inflammatory cytokines (114, 115), but these cytokines also have the ability to modify responses of other immune cell populations (115, 117).

As mentioned earlier, cortical demyelination in a subgroup of MS patients is associated with ectopic B cell follicles in the meninges which implies that B cells may be involved in cortical injury by secreting cytotoxic factors (63). *In vitro* studies using B cells from RRMS patients substantiate that they are capable of killing oligodendrocytes and neurons in an antibody-independent manner involving apoptosis (118, 119), while the identity of the cytotoxic products remains to be clarified.

However, it may also be necessary to note that the beneficial effects of anti-CD20 therapy in MS patients cannot solely be attributed to the depletion of B cells but rather CD20^+^ T cells may also be targeted (120). Although CD20 is a hallmark cell surface marker of B cells, a proportion of CD3^+^ T cells also expresses this marker (121) which are found in an increased number in the peripheral blood and CSF of MS patients (122). While it has been proposed that T cells present in the blood may acquire CD20 from B cells by a process called trogocytosis and are therefore CD3^+^CD20^+^, Schuh et al. have elaborately demonstrated that indeed a subset of T cells transcribes CD20 but no other molecules typically found on B cells (120). CD20 expressing T cells have been reported to be a highly activated pro-inflammatory cytokine-producing cell population with pathogenic potential (120, 121). Furthermore, several studies have elaborately demonstrated that this population of CD20^+^ T cells can be effectively depleted by rituximab and ocrelizumab in patients with RRMS (122–124) suggesting that depletion of this cell population might be an important consideration in the overall clinical effectiveness of anti-CD20 directed therapies (125).

### Animal Model(s) of MS: Experimental Autoimmune Encephalomyelitis (EAE)

There are of course limitations of studying the pathomechanisms of disease development in human subjects. Scientists have therefore turned to using EAE, which is one of the best characterized and most frequently used animal models for studying neuroinflammation in the human disease MS. A wide range of EAE models have been induced in a number of different species (including rats, mice, and primates) with varying degrees of efficacy to study different aspects of MS pathogenesis (126–129). Yet, most of these models are biased towards a CD4^+^ T cell-restricted immune response and no single experimental model covers all the immunological and pathological features of the human disease (130, 131). In particular, some aspects of MS, especially the progressive stage of MS, have so far been poorly covered in commonly used experimental rodent models.

As discussed above, there is a growing appreciation of the involvement of B cells in the later stage of MS where aggregates of B cells have been found in the leptomeninges of SPMS patients (81, 83). These B cell aggregates feature a complex follicle-like structure and are most likely instrumental in strong meningeal inflammation. Modeling this B cell aspect of the human disease in the conventional EAE models has yielded varying results between the different strains of rodents and with regard to the immunizing antigen(s) (85, 132).

One of the more robust mouse models that is both B cell- and antibody-dependent on the C57BL/6 background is the MP4-induced EAE (133). MP4 is a fusion protein that consists of the human isoform of myelin basic protein (MBP) and the three hydrophilic domains of proteolipid protein (PLP). Using this model several studies have successfully demonstrated both antibody-dependent and -independent roles of B cells in EAE (which mirrors aspects of the human disease as well). This includes induction of demyelination through complement activation (76, 134) and a pathogenic role for antibodies (133, 135). Of interest, B cell infiltrates are also present in the spinal cord, brain and cerebellum of MP4-immunized mice (136). In particular, aggregation of B cells that acquired features of lymphoid tissue in the chronic disease stage was detected in the cerebellar parenchyma. A detailed characterization of these B cell aggregates in MP4-induced EAE revealed that the lymphoid structures in MP4-induced EAE were segregated into a B cell and T cell zone, which is similar to secondary lymphoid tissues where B cells reside in the follicles and T cells in the parafollicular zone. Furthermore, in MP4-induced EAE, high endothelial venules (HEVs) expressing the addressins CCL19 and CCL21 were also detected in addition to the chemoattractant CXCL13 (83, 137). Heavily proliferating B cells were also found indicating recent and clonal activation (137, 138). Collectively, these findings from the MP4-induced EAE model support a strong role for B cells in MS that is not only restricted to their antibody secreting ability. While the limited availability of human tissue in conjunction with the fact that autopsied brain tissue of MS patients only provides a “snapshot,” this B cell-dependent EAE model can be exploited to answer a number of disease relevant questions. For example, time course experiments on the development of B cell follicles and studies to investigate whether B cells play a different role depending on the disease stage at which they are found can be demonstrated using this EAE model.

### Role of B Cells in MS: Lessons From Rodent Models of EAE

Studies done in other EAE models have also revealed some important aspects of B cell involvement in disease progression and pathogenesis with some of the examples mentioned below.

As mentioned earlier, B cells can function as effective APCs especially when they recognize the same antigen as T cells (139). This antigen presenting capacity of B cells has been highlighted in different B cell-dependent EAE-based studies. In EAE induced by recombinant myelin oligodendrocyte glycoprotein (rMOG) protein, activated B cells have been shown to serve as APCs that promote the differentiation and proliferation of Th1 and Th17 cells. Accordingly, anti-CD20-mediated depletion of B cells inhibited B cell-dependent activation of pathogenic T cells contributing to the overall reduction of CNS inflammation (140). Furthermore, using an adoptive transfer model of EAE, it has been demonstrated that the development of autoimmune attacks within the CNS is facilitated by induction of MHC class II on B cells followed by pathogenic cognate interactions between B- and T cells (141). Similarly, B cell-specific MHC class II knock-out (KO) mice have been found to be resistant to rMOG-induced disease indicating that B cells provide critical cellular functions independent of their humoral involvements (142).

A more favorable role of B cells in EAE has been elaborated in mice which did not express the anti-inflammatory cytokine IL-35. These mice also lost their ability to recover from T cell-mediated EAE confirming the importance of IL-10/IL-35 secreting B cells in ameliorating disease progression (143). Regulatory roles for B cells during EAE immunopathogenesis have also been discussed by other groups (144). A recent study highlights that non-selective depletion of B cells using anti-CD20 therapy concurrently abolishes preexisting regulatory B cells which are important for limiting chronic disease progression (145). Efforts to expand our understanding of this regulatory population of B cells in improving EAE severity and reducing neuroinflammation is a current topic of interest.

**Figure 1:**
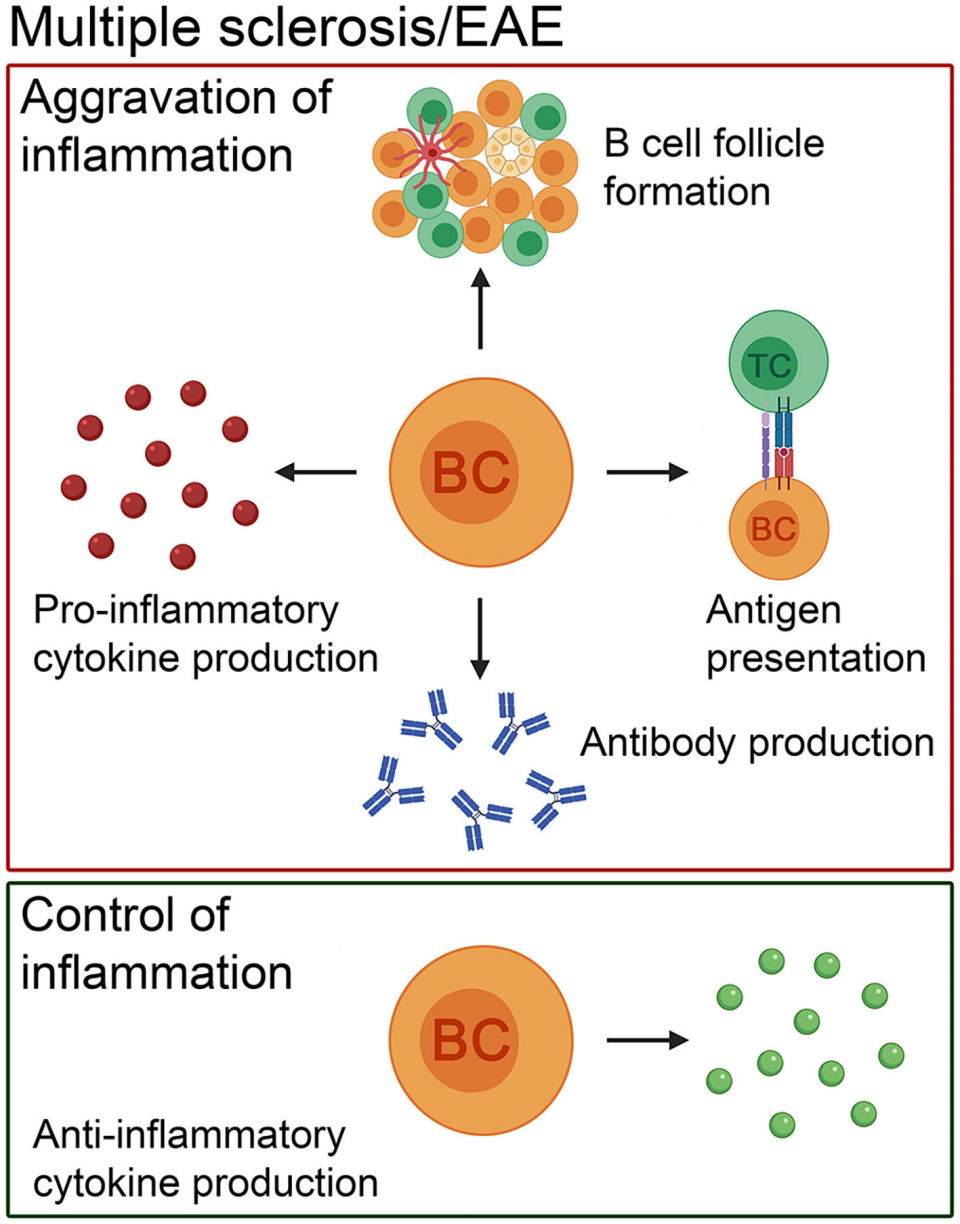
The pathogenic and beneficial effects of B cells in MS patients and in its animal model, EAE. In addition to antibody production, B cells present antigens to T cells, form ectopic lymphoid structures consisting of B- and T-cell compartments, follicular dendritic cells and high endothelial venules and produce pro-inflammatory cytokines to exacerbate the disease course. Besides the negative role of this cell population, there is evidence that B cells positively influence the disease course by secreting anti-inflammatory cytokines. BC, B cell; TC, T cell.

Taken together, there is plenty of evidence from studies done in animal models and from MS itself which repeatedly points towards a definite role of B cells in aggravating disease pathogenesis in more than one way. In addition, there is also indication of an anti-inflammatory cytokine secreting “beneficial” population of B cells in both MS patients (repopulating IL-10 secreting B cells following CD20 depletion therapy) (32) and its EAE animal model (143). Nevertheless, several unanswered questions remain including, whether the pathogenic B cell subset(s) in MS patients can be selectively depleted, based on a more detailed characterization of this cell population. Longitudinal studies to monitor changes in the pro- vs. anti-inflammatory B cell subsets in the different compartments of MS patients (or in relevant EAE models) would also provide new insights into how B cells promote or reduce neuroinflammation, respectively. Finally, it would also be interesting to explore whether these new findings can be translated into therapeutic potentials and treatment options for patients (Figure 1).

### Infectious Neuroinflammation: A Focus on Viral Diseases

As evident by the studies discussed above, the involvement of B cells as multifunctional players in MS as an example of autoimmune neuroinflammation is clear. However, as mentioned earlier, neuroinflammation can result from several other insults to the CNS which is not just restricted to being autoimmune (146). Infectious diseases of the CNS also result in neuroinflammation as an inherent host-defense mechanism to restore the normal function of the brain against the infecting pathogen (147). The importance of B cells, in general, in providing several lines of defense against a variety of pathogens and the ability of antibodies as their effector molecules in eliminating viral particles is very well established and has been discussed elsewhere (148–151). Therefore, neuroinflammation can be considered a common denominator between these two infectious and autoimmune triggers where B cells play a significant role. It is also intuitive that the “gaps” in our understanding of the contribution of B cells in autoimmune neuroinflammation, as discussed earlier, can be compensated by drawing parallels between the findings in infectious and autoimmune neuroinflammation with a focus on the involvement of B cells in both cases.

In that context, we discuss a few examples of viral infections of the CNS where B cells have been indicated to play a role in either clearing of the pathogen or progression of the infection. Furthermore, we have included examples of viral infections that are particularly relevant for MS. However, details of B cell activation in the different types of viral infections related to the CNS is beyond the scope of this review.

### Viral Infections of the CNS

Viral infections of the CNS are the most prevalent cause of encephalitis, meningitis as well as meningoencephalitis and the number of cases surpasses all bacterial, fungal, protozoal infections combined (152, 153). Following viral infections of the CNS, inflammation can occur in different anatomical regions including the meninges, brain parenchyma, the spinal cord or simultaneously in multiple regions. Examples of viral infections affecting the CNS include herpes simplex virus, adenoviruses, arboviruses, flaviviruses, and enteroviruses (154). The complexity of these viral infections is influenced by a number of different factors including the tropism of the viruses, their routes of CNS entry as well as the overall “health” of the immune system (152).

John Cunningham virus (JCV) is an important example to learn about the interplay between opportunistic viral replication and the adaptive immune system. JCV infection of the CNS is associated with improper functioning of the adaptive immune system with relation to both the B- and T cell compartments (155). The occurrence of progressive multifocal leucoencephalopathy (PML), an oftentimes deadly demyelinating disease caused by JC virus replication in the brain, has been linked to immunomodulatory treatments in patients with autoimmune diseases (156), and is also observed in immunocompromised individuals or those with hematological malignancies. B cells appear to play a complex role in mediating disease pathogenesis of PML because on the one hand, they represent a potential reservoir for JCV and on the other hand they likely play a role in the control of the infection (157). Evidence from clinical studies and those done in animal models suggests that B cells not only influence the T cell response through cytokine secretion but are also able to mount an effective humoral response against the virus which together allows the control of infection (157). Although being a very rare event, the occurrence of PML has been linked to anti-CD20 depletion therapies, indicating a potential importance of B cells in controlling JCV infection (155, 158). In general, it has been suggested that profound perturbation of B cell homeostasis by anti-CD20 therapies (as in the case of rituximab) could contribute to the development of PML (155). For instance, following anti-CD20 depletion the reconstituted B cell pool is mostly considered to be IL-10^+^ (112, 115), with IL-10 being an anti-inflammatory cytokine that suppresses both T cell- and innate cell-mediated inflammatory responses. Whether this change in the overall B cell cytokine profile together with the phenotype of the newly appearing B cells aggravates the pathogenesis of PML is a current topic of investigation (155). Nevertheless it is important to stress that anti-CD20 therapy associated PML in MS patients is an extremely rare complication compared to treatment with, for instance, natalizumab (156).

### Animal Models of Virus-Induced Neuroinflammation

Owing to the limited availability of patient material, the involvement of B cells in most viral infections of the CNS comes from the relevant animal models (159, 160). Through these various models it becomes well-established that B cells can play both detrimental as well as beneficial roles during CNS infection with encephalitic RNA viruses, such as Sindbis virus (SINV), Semliki forest virus (SFV), West Nile virus (WNV), neurotropic coronavirus, and murine cytomegalovirus (MCMV).

For instance, infection of mice with SFV suggests that brain infiltrating B cells contribute to myelin injury in SFV encephalomyelitis in both an antibody-dependent and -independent manner (161).

Extending the role of B cells beyond their capacity to modulate T cell functions, the study by Mutnal et al. is of note, where the authors demonstrate a distinctive subset of CD5^+^ B regulatory cells to infiltrate brains of mice chronically infected with MCMV. This population of regulatory B cells was found to control macrophage-dependent pro-inflammatory responses while absence of this cell population resulted in exacerbated T cell-mediated neuroinflammation post viral infection (162). In another mouse model using attenuated rabies virus it has been shown that the production of rabies-specific antibody by CNS tissue infiltrating B cells is essential for the complete elimination of the virus (163). Furthermore, studies done in mice infected with WNV have shown that B cells are critical in providing defense against early spread of infection in these mice as well as limiting infection in the CNS (164).

While the data mentioned above highlight some of the dual functions that B cells play in virus-induced neuroinflammation, numerous studies using viral models have focused on specific chemotactic signatures that allow B cell migration into the CNS.

Infection of the CNS with the neurotropic strain of mouse hepatitis virus (JHMV) in a murine model results in an acute CNS inflammatory response containing B cells (165). Antibody secreting cells were directed toward the CNS in a virus-induced chemotactic manner where CXCL9 and CXCL10 were identified as two such chemokines induced by JHMV (165). On the other hand, CXCR3 has also been identified as a chemokine receptor recruiting plasmablasts to the CNS in the same murine viral model (166). In response to another viral strain, SINV, a similar trend was noticed where CXCL13 and CCL19 were induced in the brains of mice infected with the virus (167). Similarly, during MCMV infection of the brain, CD19^+^ B cells isolated from the brain expressed chemokine receptors CXCR3, CXCR5, CCR5, and CCR7 (168). Overall, results from the different animal models of viral infections suggest CNS infiltrating B cells during viral infection migrate into the CNS in a CXCR3-, CXCR5-, and CCR7-dependent manner whose ligands are also upregulated within the CNS (169).

While most of the above-mentioned studies highlight CXCL13 (the ligand for CXCR5) as an essential B cell chemotactic factor, interestingly, during coronavirus encephalomyelitis infection in mice, naïve and early activated IgD^+^ B cells were able to migrate into the CNS independent of CXCL13-driven signals (170). This of course suggests a more complex chemokine kinetics over the course of an infection representing several possible “windows of trafficking” for B cells into the CNS. Accordingly, the subset and phenotype of B cells which migrate into the CNS may change depending on the time point.

**Figure 2:**
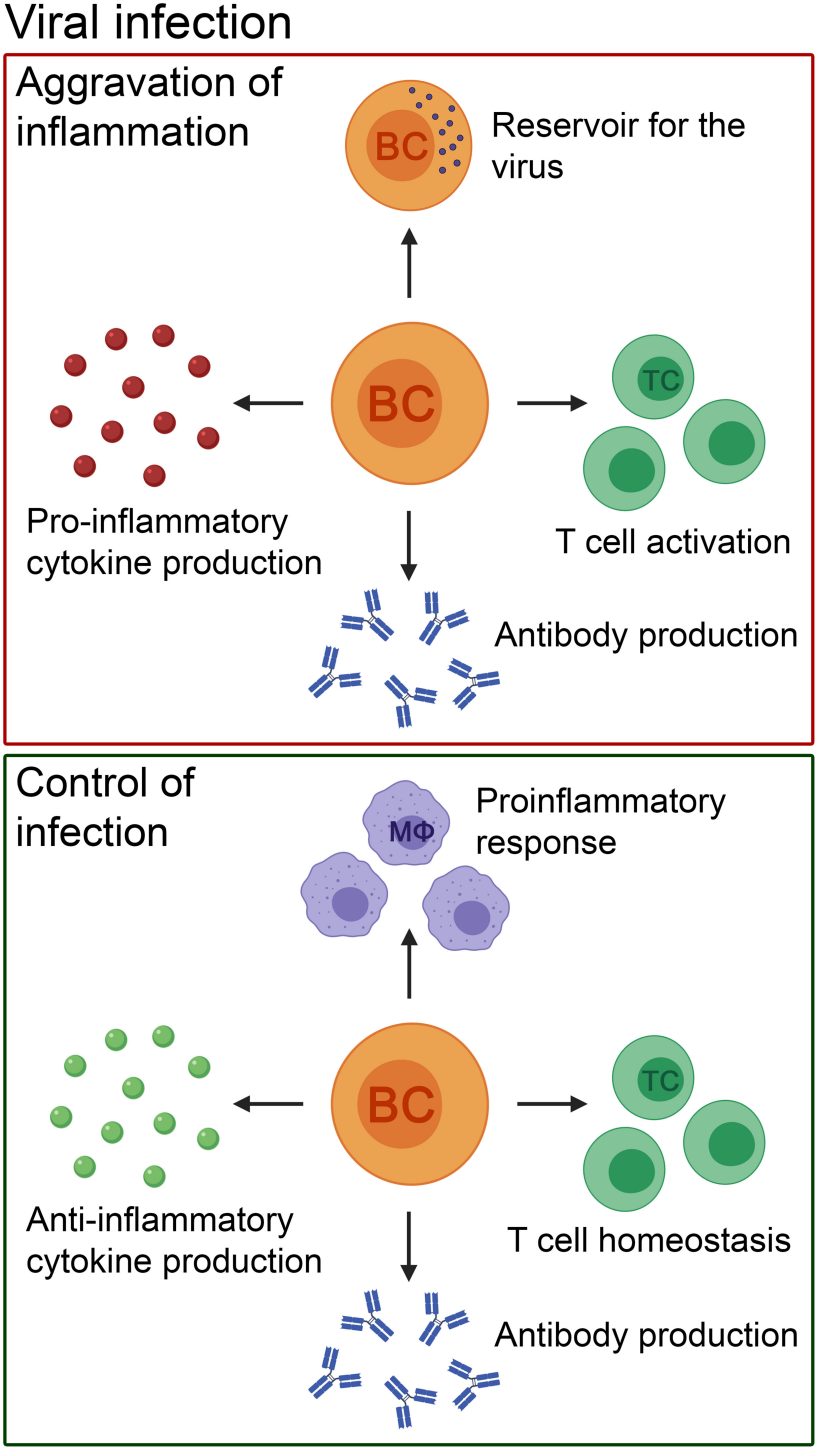
The pathogenic and beneficial effects of B cells in patients with viral infections of the CNS and their corresponding animal models. B cells can cause aggravation of inflammation by pro-inflammatory cytokine and antibody production. In addition, they can act as a reservoir for the virus, and activate T cells. Nevertheless, the role of B cells in viral infections is heterogenous. B cells control the infection by producing anti-inflammatory cytokines and antibodies to eliminate the virus. Furthermore, they have a positive effect on other immune cells by promoting T cell homeostasis and controlling innate immune cell-mediated pro-inflammatory responses (e.g., by macrophages). BC, B cell; TC, T cell; MΦ, macrophage.

Not only do viral models of inflammation give clues on migration patterns of B cells into the CNS but studies done in different viral models also present evidence that the CNS provides the necessary signals, including the expression of B-cell activating factor (BAFF), for sustained B cell viability and maintenance of a repertoire of virus-specific antibody secreting cells within the CNS (165, 168, 171). Additionally, an increased expression of BAFF mRNA in the CNS also coincides with long-term maintenance of virus-specific antibody secreting B cells in the brain (167). Sustained local antibody secretion by already infiltrated B cells in the brain seems to be an effective strategy in case of chronic viral infections of the CNS since the passage of antibodies from the periphery through intact brain barriers is insufficient (168). Another example of the CNS fostering B cell survival and differentiation comes from the Theiler's murine encephalomyelitis virus-induced demyelinating disease (TMEV-IDD) model. TMEV-IDD induced by injecting a virus into susceptible mice strains captures several aspects of chronic inflammation as seen in the progressive stages of MS (172). Using this model, during chronic infection, the predominant B cell phenotypes accumulating in the CNS were characterized to include isotype-switched B cells, memory B cells and antibody secreting cells. Mature and isotype-switched B cells were detected in the meninges and perivascular space and B cell relevant chemokines and tropic factors were elevated in the CNS in the absence of ectopic B cell follicles. Therefore, results from these studies revealed that the CNS has the ability to promote accumulation of isotype-switched B cells as well as intrathecal antibody synthesis independent of ectopic B cell follicle-like structures during chronic inflammation (173, 174) (Figure 2).

### Viral Infections Related to MS

On one hand, the involvement of B cells in the context of viral infections resulting in neuroinflammation can be emphasized by the examples mentioned above. On the other hand, viruses as infectious agents in the etiology of MS have been suspected for several decades (175). Here, we discuss the interplay between viral infections and MS with a special focus on Epstein-Barr virus (EBV).

### Epstein-Barr Virus (EBV)

Among infectious factors, EBV has the strongest epidemiological and serological connection to MS (176–178) and a relationship between EBV infection with the MS brain has long been explored. While some studies suggest that EBV may be responsible for breaking immune tolerance to CNS myelin antigens through molecular mimicry (179), others focus on the ability of the virus to infect and promote immortalization of antibody secreting B cell clones (180). It has also been suggested that the virus can act as a possible antigenic stimulus of lasting immune response within the CNS with a link to the presence of persisting OCBs (181).

EBV is a ubiquitous B-lymphotropic virus with the ability to infect, activate and latently persist in B cells for the lifetime of the infected individual (182). Furthermore, EBV is known to drive an infected B cell out of its resting state to become activated into a B cell blast and eventually become a memory B cell that can circulate in the blood (182). Suggestions have been made that when EBV-infected B cells from the periphery migrate into the CNS, they play a crucial role in propagating CNS-compartmentalized neuroinflammation (183, 184). Given that the general opinion for development of MS pathology is thought to involve interactions between T- and B cells, whether EBV-infected B cells can also activate T cells in the periphery is an attractive hypothesis (183).

Elaborate histopathological evidence demonstrating a direct link between EBV and B cells comes from the work of Aloisi and others (185) where they repeatedly identified the presence of EBV-infected B cells “exclusively” in the brain of MS patients (180, 181, 186) and not in corresponding control patients. In particular, areas with heavy B cell infiltrates have been identified as major sites of viral persistence (186).

Interestingly, a link between EBV infection and induction of human endogenous retoviral proteins on B cells has also been made (187). For example, the activation of the human endogenous retrovirus (HERV) has been suggested to be made in the presence of EBV infection where high quantities of HERV-W proteins are said to be expressed on the surface of B cells in patients with active MS (188).

**Figure 3:**
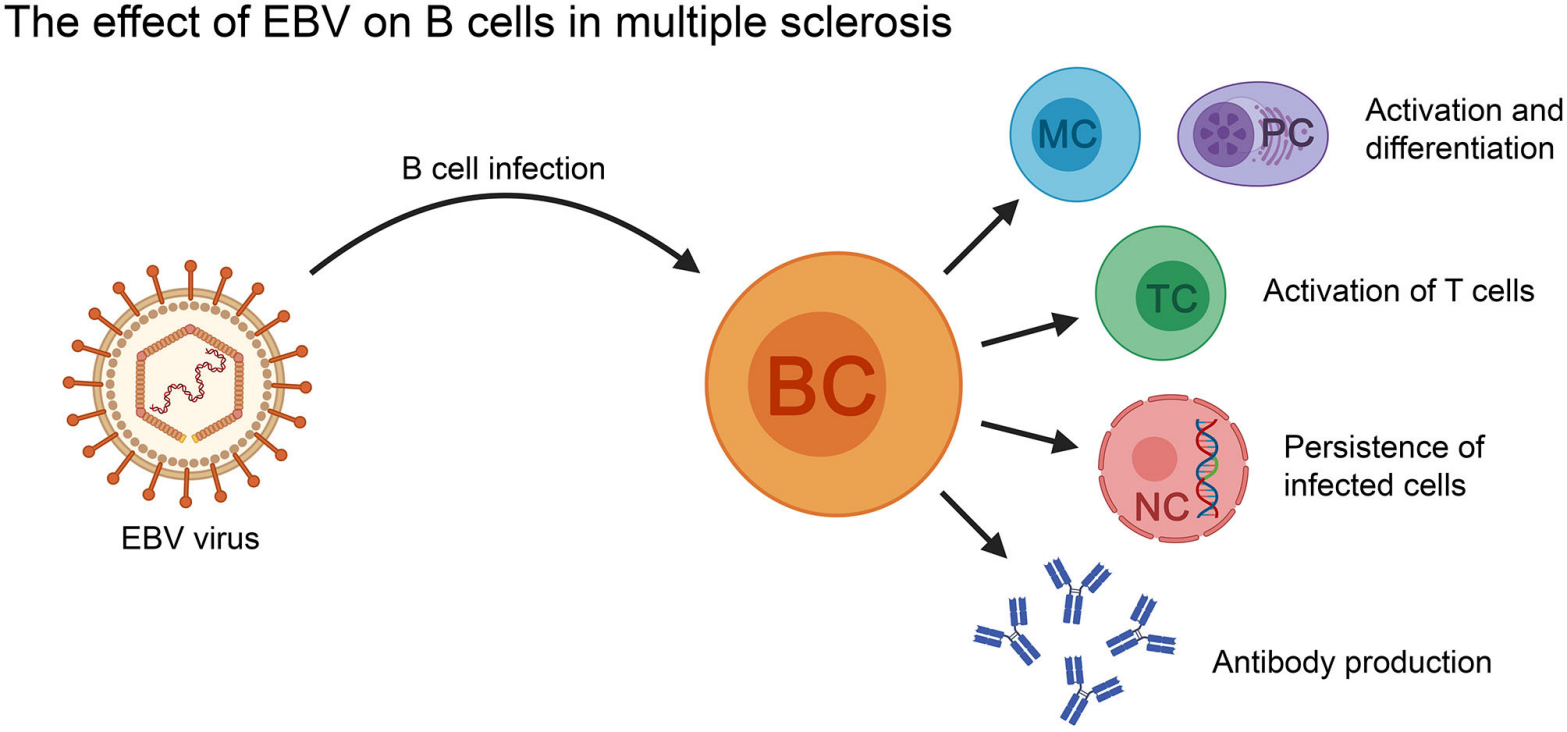
The effect of EBV on B cells in the context of MS. EBV-infected B cells can activate T cells, as well as differentiate into memory B cells and antibody secreting plasma cells (PC). The virus can persist in the infected B cells for the lifetime of the patient. BC, B cell; MC, memory B cell; NC, nucleus; PC, plasma cell; TC, T cell.

Nevertheless, it is important to note that other studies have failed to establish any relationship between EBV infection, B cells and MS (189, 190) leaving this question to what extent (if at all) EBV might be involved in MS open-ended (Figure 3).

### Cytomegalovirus (CMV)

CMV is a latent virus that is known to cause chronic activation of the immune system (191) with the seroprevalence of CMV in the general population being between 45 and 100% (192). An association between CMV infection and MS risk has been made in the past but with inconsistent results (193, 194).

Nevertheless, most studies have found that CMV seropositivity is negatively associated with MS (195, 196) with reports suggesting that CMV infection modulates the immune response to a regulatory type (196, 197). More recently it has been demonstrated that CMV infection regulates the distribution of B cell subsets in MS patients to a reduced pro-inflammatory phenotype (198)—a finding similar to what has been previously described in the case of chronic CMV infection (199). Another hypothesis of how CMV may result in milder MS symptoms could be that in patients that are CMV/EBV double seropositive, there is a balance in the immune response between these two viruses (196). However, in CMV seronegative patients, EBV could drive the immune system towards a more aggressive MS disease phenotype.

Other mechanisms by which CMV might influence MS pathogenesis can be *via* molecular mimicry or bystander activation (196, 200).

In summary, the interaction between different viruses and the immune system—in particular B cells—in MS patients seems complex with contradictory findings. Further longitudinal studies with larger patient cohorts and rigorous methodologies are required to unravel the relationship between viral infections and disease initiation as well as progression in MS.

### MS vs. Virus-Induced Neuroinflammation

It is reasonable to say that the basics of B cell biology remain the same independent of the trigger of neuroinflammation. Therefore, transferring the findings from one field of research to another may not only allow us to tackle the “unknown” better but also to look at the disorder from another perspective. As evident from the different studies mentioned above, several parallels can be drawn between virus-induced and autoimmune neuroinflammation. Here we discuss a few such examples.

Studies done in murine models of neurotropic viral infections indicate that B cells enter the CNS during acute viral infection with early infiltrating B cells expressing CXCR3 and CXCR5 (among others) and upregulation of the corresponding ligands in the CNS (169). A similar situation is observed in MS where CXCL12 and CXCL13 are elevated in actively demyelinating MS lesions (91, 201), fostering B cell entry into the CNS. Additional evidence suggests the chemokines CXCL10, CCL2, and CCL3 also to be involved in attracting B cells into the CNS in MS (202). Therefore, B cells in general appear to have a specific and common chemotactic signature that allows them to migrate into the CNS under neuroinflammatory conditions whether the source is infectious or autoimmune.

Moreover, if one was to apply the findings in the viral model (as mentioned above) by the group of Phares et al. (170) to MS, it is indeed plausible that there is a variation in the chemotactic factors in the CSF/serum over the course of the disease. This plausible time-dependent change in chemokines in MS patients may also affect the phenotype of B cells migrating into the CNS. However, given the difficulties of following this “range” of migration pattern of B cells into the CSF/CNS compartment in MS patients, the question remains open.

While these are a few instances of the chemotactic “signature” behind the migration of B cells into the CNS, there are also similarities between viral models and MS with respect to how the B cells may be able to establish themselves within the inflamed brain. For example, in line with findings from viral models (167), strong astrocytic expression of BAFF in MS lesions (87) supports B cell survival making them active participants of “trapped inflammation” (in the case of SPMS) (203). Enough circumstantial evidence suggests that through the expression of the necessary B cell survival factors, the MS brain creates an environment that is conducive for the retention of B cells within the CNS (87, 91, 202).

To what extent B cells can “establish” themselves within the CNS and the role(s) they play from within this compartment have been discussed using both viral models and EAE. Using the TMEV-IDD model DiSano et al. suggest that aggregates of B cells, independent of ectopic lymphoid-like follicle structures, are sufficient to drive B cell differentiation and also contribute to intrathecal antibody synthesis (173). This is an important finding because while there may not be an obvious presence of ectopic B cell follicles in all cases of progressive MS (189, 204), prominent CD20^+^ B cells infiltrates or clusters are detected in a higher percentage of MS patients (79, 189). Although the significance of these B cell clusters has not been well-discussed in the field of MS research, it might be interesting to see if these B cells also participate in similar functions as observed in the TMEV-IDD model.

**Figure 4:**
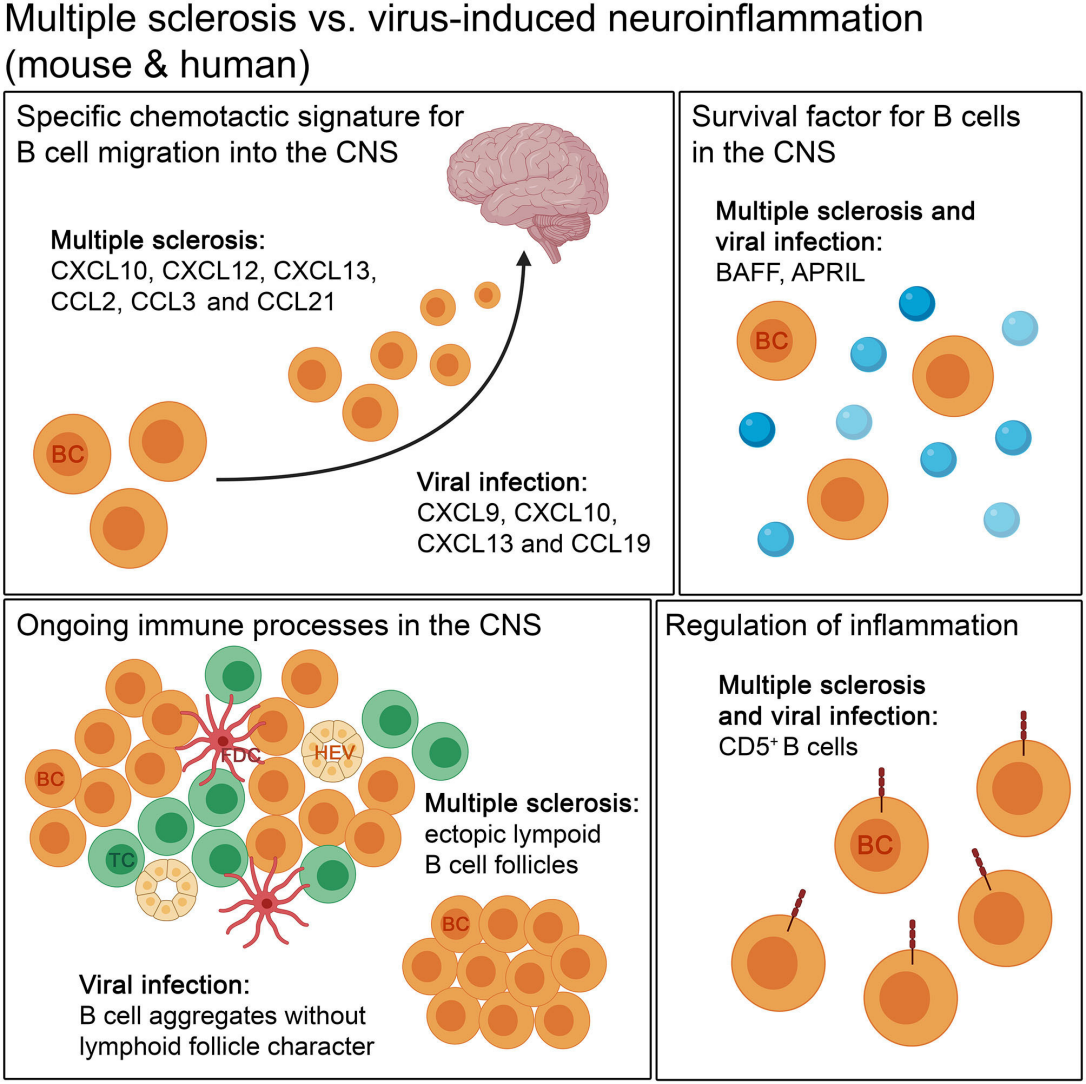
The comparison of autoimmune and infectious neuroinflammation in mouse and humans. Both diseases show a specific chemotactic signature for B cell migration into the CNS. The retention of B cells in the CNS is supported by survival factors, like BAFF and APRIL, in autoimmune and infectious neuroinflammation. B cells form aggregates during the disease course. The aggregates occurring in autoimmune diseases, like MS, can develop lymphoid follicle-like features, with compartmentalization of B cells and T cells, follicular dendritic cells and high endothelial venules, unlike in viral infection. For regulation of inflammation, CD5^+^ B cells are found in both kinds of neuroinflammation. BC, B cell; FDC, follicular dendritic cell; HEV, high endothelial venule; TC, T cell.

The significance of a specific regulatory subset of CD5^+^ B cells in the CNS following chronic viral infection has been demonstrated in animal models (162). The role of CD5^+^ B cells with a potent regulatory capacity (205, 206) has been reviewed previously (207, 208). Interestingly, clinical data from MS patients suggest that regulatory B cells with increased expression of CD5 predominantly repopulate following anti-CD20 treatment, which—when activated—secrete more anti-inflammatory IL-10 (209). Among other functions, the immunosuppressive cytokine IL-10 is also associated with T cell exhaustion allowing control of aggressive disease progression and preventing excessive tissue injury (210). Indeed, in MS, where demyelination and aggressive disease progression are associated with the presence of T cells (211), exploiting this immunoregulatory subset of IL-10 secreting CD5^+^ B cells to dampen neuroinflammation remains a current research focus (Figure 4).

## Concluding Remarks

The contribution of B cells to CNS neuroinflammatory diseases is unambiguous, as demonstrated by the examples mentioned above. One can say that the B cell response in neuroinflammation is complex and comprises a combination of both beneficial and detrimental phenotypes. Furthermore, the nature of the B cell response differs considerably between the different stages of the disease.

Following limitations of human studies, it is often necessary to extend our understanding of the involvement and importance of a particular cell type by performing experiments in appropriate animal models. Most of the animal disease models used to identify mechanisms that underlie neuroinflammation are induced artificially while mimicking all aspects of a human disease in a single animal model is not feasible. Researchers working on a particular disease often tend to use models which are within their specific area of research, while neglecting the relevance of the findings from other disease models. As an example in this review we have compared studies done in animal models of virus-induced neuroinflammation to findings in MS, although similar comparisons between other instances of neuroinflammation can also be established. It can be assumed that application of the findings from infectious neuroinflammation (with respect to the involvement of B cells) to the field of autoimmune neuroinflammation may facilitate the development of novel therapies to tackle neuroinflammatory disorders like MS and vice versa.

## Author Contributions

RC and VS outlined the subject of the review, searched for and interpreted the literature, prepared the figures, wrote the manuscript, and gave final approval of the version for publication. SK edited and revised the manuscript for important intellectual content and gave final approval of the version for publication. All figures were created with BioRender.com. All authors contributed to the article and approved the submitted version.

## Conflict of Interest

The authors declare that the research was conducted in the absence of any commercial or financial relationships that could be construed as a potential conflict of interest.
